# Detection of *Ehrlichia muris eauclairensis* in Blacklegged Ticks (*Ixodes scapularis*) and White-Footed Mice (*Peromyscus leucopus*) in Massachusetts

**DOI:** 10.1089/vbz.2022.0098

**Published:** 2023-06-05

**Authors:** Guang Xu, Erik Foster, Fumiko Ribbe, Andrias Hojgaard, Rebecca J. Eisen, Sara Paull, Stephen M. Rich

**Affiliations:** ^1^ Department of Microbiology, University of Massachusetts—Amherst, Amherst, Massachusetts, USA.; ^2^ Division of Vector-Borne Diseases, National Center for Emerging and Zoonotic Infectious Diseases, Centers for Disease Control and Prevention, Fort Collins, Colorado, USA.; ^3^ National Ecological Observatory Network, Battelle, Boulder, Colorado, USA.

**Keywords:** *Ehrlichia muris eauclairensis*, *Ixodes scapularis*, *Peromyscus leucopus*, Massachusetts

## Abstract

In 2011, *Ehrlichia muris eauclairensis* (EME) was described as a human pathogen spread by the blacklegged tick, *Ixodes scapularis*. Until very recently, its reported distribution was limited to the upper midwestern United States, mainly in Minnesota and Wisconsin. In this study, we report the detection of EME DNA in 4 of 16,146 human biting *I. scapularis* ticks submitted from Massachusetts to a passive tick surveillance program. Active tick surveillance yielded evidence of EME local transmission in the northeastern United States through detection of EME DNA in 2 of 461 host-seeking *I. scapularis* nymphs, and in 2 white-footed mice (*Peromyscus leucopus*) of 491 rodent samples collected in the National Ecological Observatory Network (NEON) Harvard Forest site in Massachusetts.

## Introduction

*E**hrlichia* spp. are the causative agents of ehrlichiosis. They are obligatory intracellular bacteria that are transmitted to vertebrates by ticks (Dumler et al., 2001). To date, six *Ehrlichia* species (*E. canis*, *E. chaffeensis*, *E. ewingii*, *E. minasensis*, *E. muris*, and *E. ruminantium*), and several Candidatus *Ehrlichia* species have been discovered (Aguiar et al., 2019; Pritt et al., 2017). Among them, *E. canis*, *E. chaffeensis*, *E. ewingii*, and *E. muris* are known to infect humans. In the United States, *E. chaffeensis* and *E. ewingii* are human pathogens transmitted by *Amblyomma americanum* ticks; *E. muris* is a human pathogen transmitted by *Ixodes scapularis* ticks in the upper midwestern United States (Pritt et al., 2017). An isolation of the “Panola Mountain Ehrlichia” has been associated with a human case of illness after the bite of a nymphal *A. americanum* from the southern United States (Loftis et al., 2008).

The type strain AS145 of *E. muris* was originally isolated from a mouse in Japan (Wen et al., 1995). In 2009, an *E. muris*-like agent (EMLA) was identified as a causative agent of human ehrlichiosis in Wisconsin and Minnesota (Pritt et al., 2011). In 2017, the Japanese isolate AS145 was proposed as *E. muris* subsp. *muris*, which is transmitted by *Haemaphysalis flava* ticks in Japan and *Ixodes persulcatus* ticks in eastern Europe. The EMLA isolates from the upper midwestern United States were proposed as a taxonomically distinct subspecies, *E. muris eauclairensis* (EME), which is associated with *I. scapularis* ticks in Minnesota and Wisconsin (Pritt et al., 2017). Previously, we reported the detection of a novel clade of *E. muris* from *Ixodes cookei* in the northeastern United States (Xu et al., 2018).

Despite the broad distribution of *I. scapularis* ticks in the United States, EME has historically been limited to the upper midwestern region of the United States. Heretofore, EME has been reported in white-footed mice (*Peromyscus leucopus*) (Castillo et al., 2015) and *I. scapularis* ticks in only three states: Wisconsin, Minnesota, and Michigan (Fleshman et al., 2022). In humans, 3 Wisconsin residents and 1 Minnesota resident out of 4247 people in 45 states tested positive for EME (Pritt et al., 2011). This study identified EME in *I. scapularis* ticks and white-footed mice (*P. leucopus*) in Massachusetts, indicating the presence of an enzootic transmission cycle of EME local to New England.

## Materials and Methods

### Collection and identification of ticks and rodents

From June 2014 to November 2020, 16,146 *I. scapularis* nymphal and adult ticks were submitted to the TickReport public testing program (https://www.tickreport.com) from Massachusetts residents and tested for *E. muris* (Table 1 and Map in Supplementary Data). *I. scapularis* ticks were first identified at the genus-level using morphologic keys, and then were differentiated from other species by a species-specific TaqMan PCR assay or by amplifying and sequencing a fragment of the tick 16S rRNA gene (Xu et al., 2019).

**Table 1.** *Ehrlichia muris eauclairensis* Samples Collected in Massachusetts

Positive sample sites in Massachusetts	Organism (tick/mouse)	Life stage	Sample size	EME positives	DNA sequences
Hardwick, Palmer, Oakham, and Petersham	*Ixodes scapularis*	Adult female	16,146	4 (0.025%)	*gltA* and *groEL* genes
NEON Harvard Forest	*I. scapularis*	Nymph	461	2 (0.434%)	*groEL* and *rrs-IGS* genes
NEON Harvard Forest	*Peromyscus leucopus*	—	491	2 (0.407%)	*gltA* and *groEL* genes

EME, *Ehrlichia muris eauclairensis*; NEON, National Ecological Observatory Network.

From 2015 to 2021, 461 nymphal *I. scapularis* ticks were tested for pathogens of the 649 total that were collected from the National Ecological Observatory Network (NEON) Harvard Forest site in Massachusetts (NEON, 2021b; NEON, 2021c), by a tick-dragging method (Table 1). A 1 square meter white cloth was dragged along the ground at a slow pace around the 160 meters perimeter of each of six sampling plots (40 × 40 meter) each year. The tick species of NEON samples were identified by a morphological characterization method at the U.S. National Tick Collection, which also archives the ticks not sent for pathogen testing.

From 2020 to 2021, 491 blood and ear samples from various rodent species were also tested for tick-borne pathogens (NEON, 2021a). These samples were collected as part of the mark-recapture effort for small mammals from the Harvard Forest & Quabbin Watershed NEON site, using Sherman live traps (Table 1). The 11,900-acre Harvard Forest site is located ∼105 km west of Boston, Massachusetts in the county of Worcester. The site is dominated by northern hardwood and coniferous forest, with some areas used for agriculture and represents a typical rural northeastern wildland, linking suburban areas outside Boston with the wildlands throughout New England (https://www.neonscience.org/field-sites/harv).

### DNA extraction and *E. muris* molecular identification

Tick and rodent samples were first sorted into individual tubes. The total nucleic acids were extracted from each sample using the Lucigen Masterpure Complete DNA and RNA Purification kit (Lucigen Corporation, Middleton, WI) following the manufacturer's protocols. A Taqman real-time PCR targeting *P13* gene was used for *E. muris* screening (Xu et al., 2018). The assay was performed in 16-μL reaction volumes using the Brilliant III qPCR Master Mix (Agilent, La Jolla, CA) in a CFX96 Touch Real-Time PCR Detection System. The reaction contained 8-μL Master Mix, 200 nM forward primer (TACCTAATTCTTCTCAAGAGATTCAGTTG), 200 nM reverse primer (ATGATGATACTGCGAACAACTATAAGAG), 200 nM dual-labeled probe (Cy5-ATATTGATAAAAGAGTCAGTGTTGATCCGTATGAGTTAGGGTT-BHQ), 1-μL template DNA, and water up to 16 μL.

An internal control was used for checking tick DNA quality. Cycling conditions included an initial activation of the Taq DNA polymerase at 95°C for 10 min, followed by 40 cycles: 95°C for 15 s and 60°C for 1 min. *E. muris* positivity of the samples was confirmed by amplifying and sequencing the citrate synthase (*gltA*) and heat shock protein (*groEL*) genes (Telford et al., 2011). DNA from EME positive tick samples (*N* = 2, *I. scapularis* nymphs) collected from the NEON Harvard Forest site through drag sampling were sent to CDC, Fort Collins, for verification and additional testing. A previously described multiplex PCR amplicon assay was utilized for testing and verification, consisting of two primer sets 859–860 (*groEL*), and 2149–2150 (*rrs-IGS*) (Hojgaard et al., 2022).

### Ethical statement

All animal protocols have been approved by Battelle's Institutional Animal Care and Use Committee (IACUC).

## Results

Four *E. muris* positive adults were identified from 16,146 *I. scapularis* ticks (0.025%) submitted to TickReport from Massachusetts from June 2014 to November 2020. The first *E. muris* positive tick was submitted from Hardwick, Massachusetts in 2017. Three more *E. muris* positive ticks were later submitted from Palmer, Oakham, and Petersham, Massachusetts in 2019 and 2020. Surprisingly, the *gltA* and *groEL* gene sequences (Supplementary Data) of these samples were identical to EME and all four ticks were adult female *I. scapularis* collected from people residing in Massachusetts without a travel history outside of Massachusetts.

From 2015 to 2021, 461 nymphal *I. scapularis* ticks collected from the NEON Harvard Forest site in Massachusetts were tested for *E. muris*. Two nymphs (0.434%, 1 in 2017 and 1 in 2019) were positive for *E. muris* (NEON, 2021b) using both the Taqman PCR and NGS sequencing assays described earlier. The *E. muris groEL* and *rrs-IGS* sequences from these two samples were identical to each other, as well as to the EME control sample (Supplementary Data). In addition to tick samples, 2 *P. leucopus* blood samples (0.407%) were positive for *E. muris* of the 491 rodent blood or ear samples collected from the same site in 2020 and 2021 (NEON, 2021a). EME may have different abundances in blood and tissues. All obtained *E. muris* sequences of the *gltA* and *groEL* genes from ticks and rodents were identical to each other, as well as to EME.

## Discussion

Since its discovery in 2009 (Pritt et al., 2011), EME has been thought to be localized in the upper midwestern United States. It has been detected in *I. scapularis* ticks, strictly in Minnesota (Johnson et al., 2018), Wisconsin (Pritt et al., 2011; Telford et al., 2011), and Michigan (Fleshman et al., 2022). EME positive samples were also detected in *P. leucopus* mice from Minnesota and Wisconsin (Castillo et al., 2015) and human blood from five states: Indiana, Michigan, Minnesota, North Dakota, and Wisconsin (Dahlgren et al., 2016).

This study identified EME in two nymphal *I. scapularis* ticks collected from the NEON Harvard Forest site in Massachusetts and four adult *I. scapularis* ticks from people residing in Massachusetts without a travel history. More importantly, two *P. leucopus* mice collected from the NEON Harvard Forest site were also positive for EME. These data greatly expand the known geographic distribution of the pathogen, indicating that the number of states reporting EME prevalence might be underestimated. Similar to previous studies in the Upper Midwest (Castillo et al., 2015; Johnson et al., 2018; Pritt et al., 2011), *I. scapularis* ticks and *P. leucopus* mice also appear to be important in maintaining the enzootic cycle of EME in Massachusetts.

In general, the prevalence of EME in ticks appears to be low. Only two (0.026%) EME positives were found in 7800 human-biting *I. scapularis* ticks from 33 states in the northeastern, midwestern, and southeastern regions (Xu et al., 2018). However, the reported infection rate in the upper midwestern United States was higher: EME was detected in 17 of 697 (2.4%) *I. scapularis* ticks collected in Wisconsin and Minnesota (Pritt et al., 2011), and 2 of 36 (5.6%) adult ticks in Wisconsin (Stauffer et al., 2020). The prevalence of EMLA infection was 0.94% from 760 *I. scapularis* adult ticks collected in Wisconsin between 1992 and 1997 (Telford et al., 2011), and 4.6% of 196 *I. scapularis* ticks removed from soldiers in Wisconsin and 0.5% of 365 ticks in Minnesota for a 15-year period (Stromdahl et al., 2014).

Johnson et al. (2018) reported EME in 13 of 64 sampling sites throughout Minnesota with an estimated nymphal infection prevalence rate of 1.29% (95% CI: 0.77–2.04). Blood analysis of 75,007 patients collected from 2004 to 2013 from 50 states found that 69 patients (0.1%) were positive for EME (Dahlgren et al., 2016). Unlike Lyme disease agents in *I. scapularis* ticks, the prevalence of EME is also low in Massachusetts. Only four EME (0.025%) positives were found from 16,146 *I. scapularis* ticks submitted from Massachusetts. In the NEON Harvard forest site, the prevalence of EME was 0.434% in nymphal *I. scapularis* ticks and 0.407% in rodents.

Although a mouse model for ehrlichiosis caused by EME has been developed (Saito et al., 2015), the natural animal reservoir of this pathogen is largely unknown and probably involves small rodents, such as *Microtus agrestis* and *Myodes glareolus* in Western Siberia and *Eothenomys kageus* in Japan. The white-footed mouse, *P. leucopus*, is considered an important host for nymphal *I. scapularis* and a reservoir of EME (Pritt et al., 2011). Interestingly, the woodland deer mouse, *Peromyscus maniculatus*, was likely responsible for feeding and infecting more ticks with pathogens (including EME) than *P. leucopus* in the upper midwestern United States (Larson et al., 2021).

**FIG. 1.**
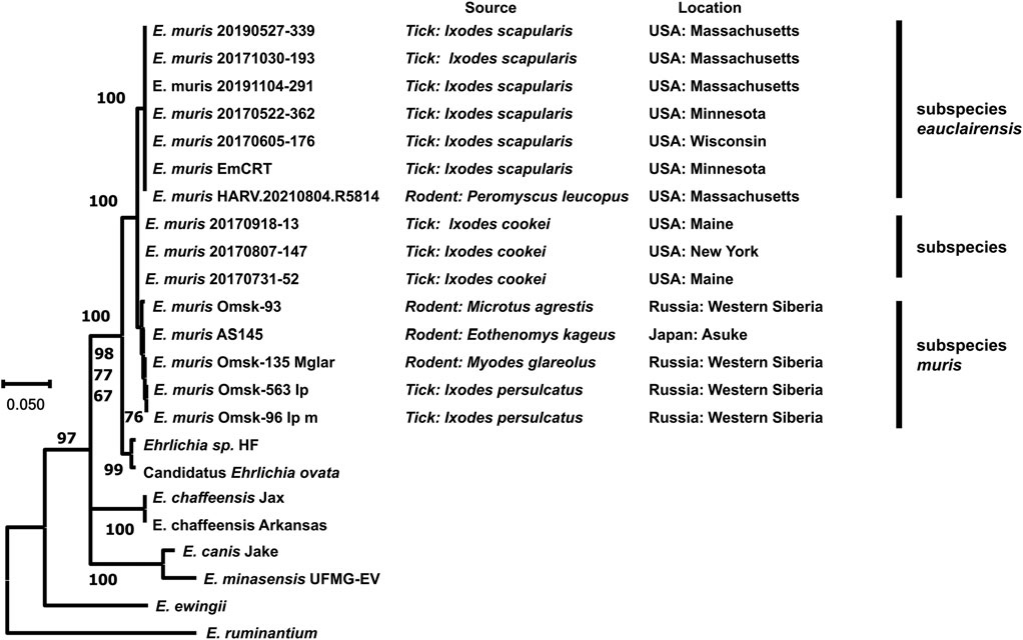
Phylogenetic tree of Ehrlichia citrate synthase (*gltA*) and heat shock protein (*groEL*) genes constructed by the maximum-likelihood method of MEGA11 software. The total length of two concatenated genes is 1045 bp. Hasegawa–Kishino–Yano with invariable sites was selected as the best model based on Bayesian information criterion scores. Numbers on the branches represent bootstrap support with 500 bootstrap replicates. Scale bar indicates nucleotide substitutions per site.

Although *P. maniculatus* has been found in Berkshire, Franklin, and Hampshire counties in Massachusetts, the reservoir competence of *P. maniculatus* to EME is unknown in this region. Moreover, there is a distinct *E. muris* clade in *I. cookei* ticks in the northeastern United States (Xu et al., 2018). Because *I. scapularis* and *I. cookei* may feed on the same host species, the transmission cycle of two different *E. muris* subspecies can be complicated and overlapping. Further studies are needed to understand the importance of *Ixodes* species ticks as a vector and *Peromyscus* species as a reservoir of *E. muris* maintenance and transmission in the northeastern United States (Fig. 1).

It has been suggested that EME possibly evolved in midwestern *I. scapularis* populations that were geographically isolated until very recently from populations in the northeastern and southeastern United States during the Pleistocene glacial period (Fleshman et al., 2022). The finding of EME in the northeastern *I. scapularis* populations indicates three possibilities: (1) EME first evolved in the midwestern region, then was very recently transmitted from the midwestern to the northeastern United States; (2) EME was also present in the northeastern *I. scapularis* populations and was not detected until this study due to the low pathogen prevalence and lack of large-scale surveillance; or (3) EME was present at low prevalence at the time the midwestern and northeastern populations were geographically isolated and it has evolved in these populations, but went largely undetected due to limited testing with specific molecular detection assays.

A retrospective analysis on *Ixodes* ticks and human samples from Massachusetts may help us to better understand the transmission history and rate of spread of EME in New England. Although >115 ehrlichiosis cases caused by EME have been identified in patients in the Upper Midwest since 2009 (https://www.cdc.gov/ehrlichiosis/stats/index.html), no EME-associated cases of human ehrlichiosis have been reported in Massachusetts. The results of this study indicate that the known presence and prevalence of EME might be underestimated. In 1994, an ehrlichiosis case and a substantial frequency of seropositivity for *Ehrlichia* were reported among residents on Cape Cod (Rynkiewicz and Liu, 1994), where *A. americanum* populations were not established until the 2010s (Telford et al., 2019). Alternatively, this ehrlichiosis case may relate to *Anaplasma phagocytophilum* due to cross-reactivity among antigens of *Ehrlichia* and *Anaplasma*.

We found identical DNA sequences of EME samples in Massachusetts and the upper midwestern. However, little is known about the possible linkage between the low genetic diversity and EME's pathogenicity and host specificity. Further study is warranted to better understand the vector competence, the natural enzootic cycle, and the ecological niche of EME. The potential range in New England should also be monitored. Most importantly, human ehrlichiosis should be considered as a possible diagnosis for tick bite victims in New England.

## Supplementary Material

Supplemental data
