# Supplementary material for: Detection of Ehrlichia muris eauclairensis in Blacklegged Ticks (Ixodes scapularis) and White-Footed Mice (Peromyscus leucopus) in Massachusetts
Source: Vector Borne Zoonotic Dis. 2023 Jun 5;23(6):311–5. doi: 10.1089/vbz.2022.0098 (PMC10278004; doi:10.1089/vbz.2022.0098)
Supplement: Supplemental data [file Supp_Data.docx]

Detection of *Ehrlichia muris* *eauclairensis* in blacklegged ticks (*Ixodes scapularis*) and white-footed mice (*Peromyscus leucopus*) in Massachusetts

Guang Xu^1^, Erik Foster^2^, Fumiko Ribbe^1^, Andrias Hojgaard^2^, Rebecca J. Eisen^2^, Sara Paull^3^, Stephen M. Rich^1^

1: Department of Microbiology, University of Massachusetts – Amherst, Amherst, MA 01003

2: Division of Vector-Borne Diseases, National Center for Emerging and Zoonotic Infectious Diseases, Centers for Disease Control and Prevention, Fort Collins, CO 80521

3: National Ecological Observatory Network, Battelle, 1685 38^th^ St, Boulder, CO 80301, USA,

Location map of the NEON Harvard forest site


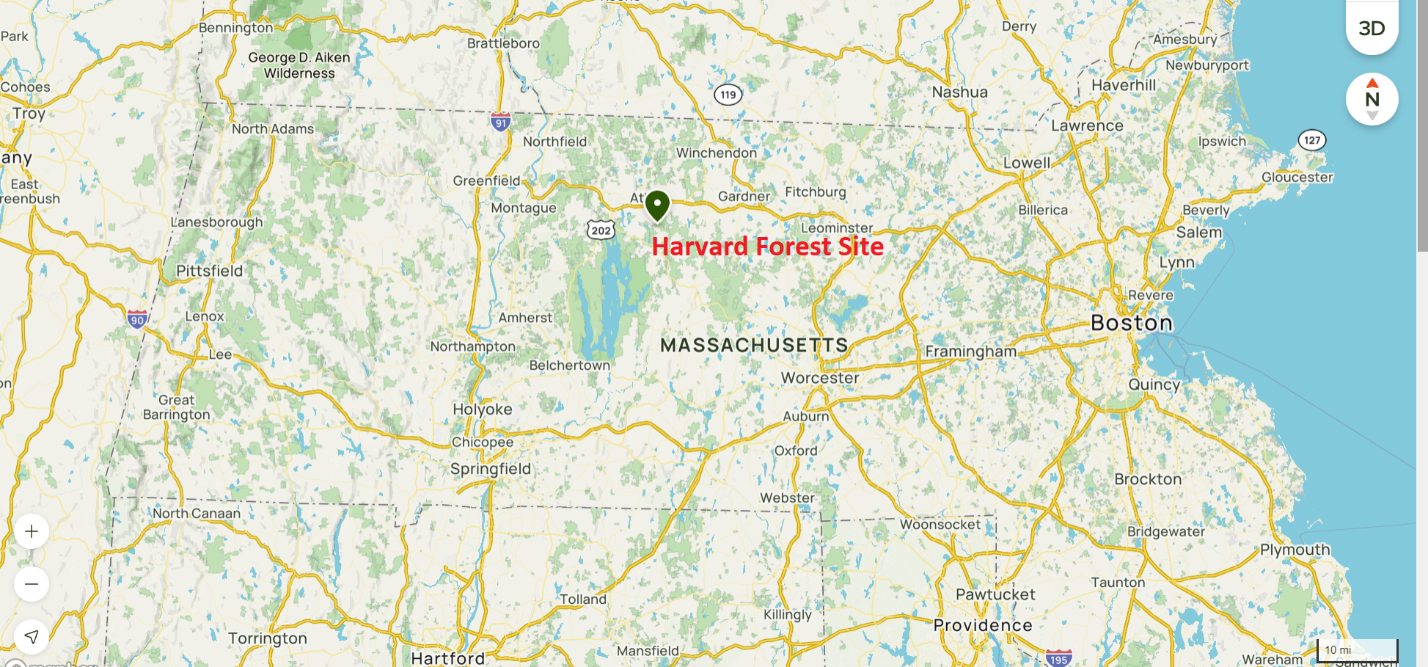


DNA sequences:

>Ehrlichia muris isolate 20171030-193 citrate synthase gene, partial cds, Ixodes scapularis adult female on human from Hardwick Massachusetts

TGGCATGTTTTTCTGCCTTAGCATCATATTATCATGATCAAGAAGCTGATAAAGATGAATTAAAGTATGCTAAATTAGCTGTGGCTAAAATTGCAAGCATAGTAGCGCTGATTTATAGATATATAACGAATCAGGATTTTATTACAGCTGATACAAAATTGTCTTACAGTGAAAATTTCGTGCATATGATGTTTGATATTTCTTCCTATAAATTCACTCAGGTTGTTGCTAAAGCTTTGGATATTATCTTTGTATTGCATGCTGATCATGAGCAAAATGCTTCTACTGCTACTGTTAGGTTAGCTGGTTCTTCAGGAGCAGATTTATTTGCTTGTTTAGTAGCAGGAACAGCAACTTTATGGGGTCCAGCTCATGGTGGAGCAAATGAAGCTGTAATTAATATGTTAATGACAATTGAAAAACCAAGTAATGTAAAACAGTTTATTCAAAAAGTGAAGGATGGTAGTAAGACTACAAGATTAATGGGTTTTGGTCA

>Ehrlichia muris isolate 20190527-339 citrate synthase gene, partial cds, Ixodes scapularis adult female on human from Palmer, Massachusetts

TGGCATGTTTTTCTGCCTTAGCATCATATTATCATGATCAAGAAGCTGATAAAGATGAATTAAAGTATGCTAAATTAGCTGTGGCTAAAATTGCAAGCATAGTAGCGCTGATTTATAGATATATAACGAATCAGGATTTTATTACAGCTGATACAAAATTGTCTTACAGTGAAAATTTCGTGCATATGATGTTTGATATTTCTTCCTATAAATTCACTCAGGTTGTTGCTAAAGCTTTGGATATTATCTTTGTATTGCATGCTGATCATGAGCAAAATGCTTCTACTGCTACTGTTAGGTTAGCTGGTTCTTCAGGAGCAGATTTATTTGCTTGTTTAGTAGCAGGAACAGCAACTTTATGGGGTCCAGCTCATGGTGGAGCAAATGAAGCTGTAATTAATATGTTAATGACAATTGAAAAACCAAGTAATGTAAAACAGTTTATTCAAAAAGTGAAGGATGGTAGTAAGACTACAAGATTAATGGGTTTTGGTCA

>Ehrlichia muris isolate 20191104-291 citrate synthase gene, partial cds, Ixodes scapularis adult female on human from Oakham, Massachusetts

TGGCATGTTTTTCTGCCTTAGCATCATATTATCATGATCAAGAAGCTGATAAAGATGAATTAAAGTATGCTAAATTAGCTGTGGCTAAAATTGCAAGCATAGTAGCGCTGATTTATAGATATATAACGAATCAGGATTTTATTACAGCTGATACAAAATTGTCTTACAGTGAAAATTTCGTGCATATGATGTTTGATATTTCTTCCTATAAATTCACTCAGGTTGTTGCTAAAGCTTTGGATATTATCTTTGTATTGCATGCTGATCATGAGCAAAATGCTTCTACTGCTACTGTTAGGTTAGCTGGTTCTTCAGGAGCAGATTTATTTGCTTGTTTAGTAGCAGGAACAGCAACTTTATGGGGTCCAGCTCATGGTGGAGCAAATGAAGCTGTAATTAATATGTTAATGACAATTGAAAAACCAAGTAATGTAAAACAGTTTATTCAAAAAGTGAAGGATGGTAGTAAGACTACAAGATTAATGGGTTTTGGTCA

>Ehrlichia muris isolate 20200413-153 citrate synthase gene, partial cds, Ixodes scapularis adult female on human from Petersham, Massachusetts

TGGCATGTTTTTCTGCCTTAGCATCATATTATCATGATCAAGAAGCTGATAAAGATGAATTAAAGTATGCTAAATTAGCTGTGGCTAAAATTGCAAGCATAGTAGCGCTGATTTATAGATATATAACGAATCAGGATTTTATTACAGCTGATACAAAATTGTCTTACAGTGAAAATTTCGTGCATATGATGTTTGATATTTCTTCCTATAAATTCACTCAGGTTGTTGCTAAAGCTTTGGATATTATCTTTGTATTGCATGCTGATCATGAGCAAAATGCTTCTACTGCTACTGTTAGGTTAGCTGGTTCTTCAGGAGCAGATTTATTTGCTTGTTTAGTAGCAGGAACAGCAACTTTATGGGGTCCAGCTCATGGTGGAGCAAATGAAGCTGTAATTAATATGTTAATGACAATTGAAAAACCAAGTAATGTAAAACAGTTTATTCAAAAAGTGAAGGATGGTAGTAAGACTACAAGATTAATGGGTTTTGGTCA

>Ehrlichia muris isolate 20171030-193 groEL gene, partial cds, Ixodes scapularis adult female on human from Hardwick Massachusetts

AAGGGATTCAAAGAATTGGATGTTGAAAAAACTGATGGTATGCAGTTTGATCGTGGTTACCTTTCTCCTTATTTTGTAACCAACTCAGAGAAGATGTTGGTGGAATTTGAAAATCCTTATATCTTACTAACAGAGAAAAAGCTTAATATAATACAGCCTATATTACCAATTTTAGAAAATGTGGCTAGATCAGGAAGACCTCTTTTAATTATTGCAGAGGATGTAGAAGGAGAAGCGCTTAGTACTCTTGTTTTGAATAAATTACGTGGGGGGTTACATGTAGCAGCAGTTAAAGCACCAGGATTTGGGGATAGAAGAAAAGATATGTTAGGTGATATTGCTATTTTAACTGGAGCTAAGCATGTAATAAGTGATGATCTTGCAATAAAGATGGAAGATTTAACTTTAGCTGAATTAGGTACTGCTAAAAATATACGTATTACAAAAGATACTACTACTATTATTGGCAGTGTAGATAATAGTTCTACTAATGTACAAAGTAGAATTAATCAAATTAAAATGCAAATTGAAGCTTCTACTTCAGATTATGATAAAGAAAAGTTAAGAGAACGTTTGGCTAAGCTATCAGGTGGTGTTGCTGTCTTAAAGGTTGGTGG

>Ehrlichia muris isolate 20190527-339 groEL gene, partial cds, Ixodes scapularis adult female on human from Palmer, Massachusetts

AAGGGATTCAAAGAATTGGATGTTGAAAAAACTGATGGTATGCAGTTTGATCGTGGTTACCTTTCTCCTTATTTTGTAACCAACTCAGAGAAGATGTTGGTGGAATTTGAAAATCCTTATATCTTACTAACAGAGAAAAAGCTTAATATAATACAGCCTATATTACCAATTTTAGAAAATGTGGCTAGATCAGGAAGACCTCTTTTAATTATTGCAGAGGATGTAGAAGGAGAAGCGCTTAGTACTCTTGTTTTGAATAAATTACGTGGGGGGTTACATGTAGCAGCAGTTAAAGCACCAGGATTTGGGGATAGAAGAAAAGATATGTTAGGTGATATTGCTATTTTAACTGGAGCTAAGCATGTAATAAGTGATGATCTTGCAATAAAGATGGAAGATTTAACTTTAGCTGAATTAGGTACTGCTAAAAATATACGTATTACAAAAGATACTACTACTATTATTGGCAGTGTAGATAATAGTTCTACTAATGTACAAAGTAGAATTAATCAAATTAAAATGCAAATTGAAGCTTCTACTTCAGATTATGATAAAGAAAAGTTAAGAGAACGTTTGGCTAAGCTATCAGGTGGTGTTGCTGTCTTAAAGGTTGGTGG

>Ehrlichia muris isolate 20191104-291 groEL gene, partial cds, Ixodes scapularis adult female on human from Oakham, Massachusetts

AAGGGATTCAAAGAATTGGATGTTGAAAAAACTGATGGTATGCAGTTTGATCGTGGTTACCTTTCTCCTTATTTTGTAACCAACTCAGAGAAGATGTTGGTGGAATTTGAAAATCCTTATATCTTACTAACAGAGAAAAAGCTTAATATAATACAGCCTATATTACCAATTTTAGAAAATGTGGCTAGATCAGGAAGACCTCTTTTAATTATTGCAGAGGATGTAGAAGGAGAAGCGCTTAGTACTCTTGTTTTGAATAAATTACGTGGGGGGTTACATGTAGCAGCAGTTAAAGCACCAGGATTTGGGGATAGAAGAAAAGATATGTTAGGTGATATTGCTATTTTAACTGGAGCTAAGCATGTAATAAGTGATGATCTTGCAATAAAGATGGAAGATTTAACTTTAGCTGAATTAGGTACTGCTAAAAATATACGTATTACAAAAGATACTACTACTATTATTGGCAGTGTAGATAATAGTTCTACTAATGTACAAAGTAGAATTAATCAAATTAAAATGCAAATTGAAGCTTCTACTTCAGATTATGATAAAGAAAAGTTAAGAGAACGTTTGGCTAAGCTATCAGGTGGTGTTGCTGTCTTAAAGGTTGGTGG

>Ehrlichia muris isolate 20200413-153 groEL gene, partial cds, Ixodes scapularis adult female on human from Petersham, Massachusetts

AAGGGATTCAAAGAATTGGATGTTGAAAAAACTGATGGTATGCAGTTTGATCGTGGTTACCTTTCTCCTTATTTTGTAACCAACTCAGAGAAGATGTTGGTGGAATTTGAAAATCCTTATATCTTACTAACAGAGAAAAAGCTTAATATAATACAGCCTATATTACCAATTTTAGAAAATGTGGCTAGATCAGGAAGACCTCTTTTAATTATTGCAGAGGATGTAGAAGGAGAAGCGCTTAGTACTCTTGTTTTGAATAAATTACGTGGGGGGTTACATGTAGCAGCAGTTAAAGCACCAGGATTTGGGGATAGAAGAAAAGATATGTTAGGTGATATTGCTATTTTAACTGGAGCTAAGCATGTAATAAGTGATGATCTTGCAATAAAGATGGAAGATTTAACTTTAGCTGAATTAGGTACTGCTAAAAATATACGTATTACAAAAGATACTACTACTATTATTGGCAGTGTAGATAATAGTTCTACTAATGTACAAAGTAGAATTAATCAAATTAAAATGCAAATTGAAGCTTCTACTTCAGATTATGATAAAGAAAAGTTAAGAGAACGTTTGGCTAAGCTATCAGGTGGTGTTGCTGTCTTAAAGGTTGGTGG

>Ehrlichia muris isolate HARV.20210804.R5814.B citrate synthase gene, partial cds, Peromyscus leucopus collected in the National Ecological Observatory Network (NEON) Harvard Forest site in Massachusetts.

TGGCATGTTTTTCTGCCTTAGCATCATATTATCATGATCAAGAAGCTGATAAAGATGAATTAAAGTATGCTAAATTAGCTGTGGCTAAAATTGCAAGCATAGTAGCGCTGATTTATAGATATATAACGAATCAGGATTTTATTACAGCTGATACAAAATTGTCTTACAGTGAAAATTTCGTGCATATGATGTTTGATATTTCTTCCTATAAATTCACTCAGGTTGTTGCTAAAGCTTTGGATATTATCTTTGTATTGCATGCTGATCATGAGCAAAATGCTTCTACTGCTACTGTTAGGTTAGCTGGTTCTTCAGGAGCAGATTTATTTGCTTGTTTAGTAGCAGGAACAGCAACTTTATGGGGTCCAGCTCATGGTGGAGCAAATGAAGCTGTAATTAATATGTTAATGACAATTGAAAAACCAAGTAATGTAAAACAGTTTATTCAAAAAGTGAAGGATGGTAGTAAGACTACAAGATTAATGGGTTTTGGTCA

> Ehrlichia muris isolate HARV.20210804.R5814.B groEL gene, partial cds, Peromyscus leucopus collected in the National Ecological Observatory Network (NEON) Harvard Forest site in Massachusetts.

AAGGGATTCAAAGAATTGGATGTTGAAAAAACTGATGGTATGCAGTTTGATCGTGGTTACCTTTCTCCTTATTTTGTAACCAACTCAGAGAAGATGTTGGTGGAATTTGAAAATCCTTATATCTTACTAACAGAGAAAAAGCTTAATATAATACAGCCTATATTACCAATTTTAGAAAATGTGGCTAGATCAGGAAGACCTCTTTTAATTATTGCAGAGGATGTAGAAGGAGAAGCGCTTAGTACTCTTGTTTTGAATAAATTACGTGGGGGGTTACATGTAGCAGCAGTTAAAGCACCAGGATTTGGGGATAGAAGAAAAGATATGTTAGGTGATATTGCTATTTTAACTGGAGCTAAGCATGTAATAAGTGATGATCTTGCAATAAAGATGGAAGATTTAACTTTAGCTGAATTAGGTACTGCTAAAAATATACGTATTACAAAAGATACTACTACTATTATTGGCAGTGTAGATAATAGTTCTACTAATGTACAAAGTAGAATTAATCAAATTAAAATGCAAATTGAAGCTTCTACTTCAGATTATGATAAAGAAAAGTTAAGAGAACGTTTGGCTAAGCTATCAGGTGGTGTTGCTGTCTTAAAGGTTGGTGG

>2149-2150, reference DNA from culture E. muris eauclairensis (from RZB Atlanta)

TAGGCTCATCTAATAGCGATAAATCTTTCCCCCGCAGGGATTATACAGTATTACCCATCATTTCTAATGGCTATTCCATACTACTAGGTAGATTCCTATGCATTACTCACCCGTCTGCCACTAATAACTGCCTATAACAAAAAAGCTATAGGCAGTTATCCGTTCGACTTGCATGTGTTAGGCTTGCCGCCAGCGTTCGTTCTGAGCCAGGATCAAACTCTCAAGTTTGACAAACCATATATACTATGGCTTGGACTCATGCAAACAAACATAAATGCGTCAATGCATGATAAATAAATATAGCTACTATGCATCGCCGCCTTTATTTCTA

>859-860, reference DNA from culture E. muris eauclairensis (from RZB Atlanta)

TACTCAGAGTGCTTCTCAATGTAATGATAAAGTTGGTGATGGAACAACTACATGTTCTATTTTAACTGCAAAAGTTATAGAAGAAGTATCTAAAGCTAAAGCTGCTGGAGCAGATATTGTATGTATTAAAGAAGGTGTATTAAAAGCTAAAGAAGCTGTGCTAGAAGCATTAATGTCTATGAAGCGTGAAGTATTGTCTGAAGAGGAGATTGCTCAAGTTGCTACTATTTCAGCTAATGGAGATAAAAACATAGGTAGTAAAATTGCTCAATGTGTTCAAGAAGTTGGTAAAGATGGAGTTATTACAGTTGAAGAAAGTAAGGGATTCAAAGAATTGGATGTTGAAAAAACTGATGGTATGC

>2149-2150_EME_candidate_QT00000000145, Ixodes scapularis nymph collected in the National Ecological Observatory Network (NEON) Harvard Forest site in Massachusetts.

TAGGCTCATCTAATAGCGATAAATCTTTCCCCCGCAGGGATTATACAGTATTACCCATCATTTCTAATGGCTATTCCATACTACTAGGTAGATTCCTATGCATTACTCACCCGTCTGCCACTAATAACTGCCTATAACAAAAAAGCTATAGGCAGTTATCCGTTCGACTTGCATGTGTTAGGCTTGCCGCCAGCGTTCGTTCTGAGCCAGGATCAAACTCTCAAGTTTGACAAACCATATATACTATGGCTTGGACTCATGCAAACAAACATAAATGCGTCAATGCATGATAAATAAATATAGCTACTATGCACCGCCGCCTTTATTTCTA

>2149-2150_EME_candidate_LMZ-4280, Ixodes scapularis nymph collected in the National Ecological Observatory Network (NEON) Harvard Forest site in Massachusetts.

TAGGCTCATCTAATAGCGATAAATCTTTCCCCCGCAGGGATTATACAGTATTACCCATCATTTCTAATGGCTATTCCATACTACTAGGTAGATTCCTATGCATTACTCACCCGTCTGCCACTAATAACTGCCTATAACAAAAAAGCTATAGGCAGTTATCCGTTCGACTTGCATGTGTTAGGCTTGCCGCCAGCGTTCGTTCTGAGCCAGGATCAAACTCTCAAGTTTGACAAACCATATATACTATGGCTTGGACTCATGCAAACAAACATAAATGCGTCAATGCATGATAAATAAATATAGCTACTATGCACCGCCGCCTTTATTTCTA

>859-860_EME_candidate_QT00000000145, Ixodes scapularis nymph collected in the National Ecological Observatory Network (NEON) Harvard Forest site in Massachusetts.

TACTCAGAGTGCTTCTCAATGTAATGATAAAGTTGGTGATGGAACAACTACATGTTCTATTTTAACTGCAAAAGTTATAGAAGAAGTATCTAAAGCTAAAGCTGCTGGAGCAGATATTGTATGTATTAAAGAAGGTGTATTAAAAGCTAAAGAAGCTGTGCTAGAAGCATTAATGTCTATGAAGCGTGAAGTATTGTCTGAAGAGGAGATTGCTCAAGTTGCTACTATTTCAGCTAATGGAGATAAAAACATAGGTAGTAAAATTGCTCAATGTGTTCAAGAAGTTGGTAAAGATGGAGTTATTACAGTTGAAGAAAGTAAGGGATTCAAAGAATTGGATGTTGAAAAAACTGATGGTATGC

>859-860_EME_candidate_LMZ-4280, Ixodes scapularis nymph collected in the National Ecological Observatory Network (NEON) Harvard Forest site in Massachusetts.

TACTCAGAGTGCTTCTCAATGTAATGATAAAGTTGGTGATGGAACAACTACATGTTCTATTTTAACTGCAAAAGTTATAGAAGAAGTATCTAAAGCTAAAGCTGCTGGAGCAGATATTGTATGTATTAAAGAAGGTGTATTAAAAGCTAAAGAAGCTGTGCTAGAAGCATTAATGTCTATGAAGCGTGAAGTATTGTCTGAAGAGGAGATTGCTCAAGTTGCTACTATTTCAGCTAATGGAGATAAAAACATAGGTAGTAAAATTGCTCAATGTGTTCAAGAAGTTGGTAAAGATGGAGTTATTACAGTTGAAGAAAGTAAGGGATTCAAAGAATTGGATGTTGAAAAAACTGATGGTATGC
